# Smart manufacturing-driven probabilistic process planning for components via AP-BiLSTM-ATT

**DOI:** 10.3389/frai.2025.1745372

**Published:** 2026-01-12

**Authors:** Wei Yang, Jinyan Liang, Xiaoyu Zhang, Xiting Peng

**Affiliations:** 1 School of Information Science and Engineering, Shenyang University of Technology, Shenyang, China; 2 School of Computer Science and Engineering, College of Arts & Information Engineering, Dalian Polytechnic University, Dalian, China; 3 School of Artificial Intelligence, Shenyang University of Technology, Shenyang, China

**Keywords:** AP-BiLSTM-ATT, intelligent reasoning, knowledge representation, process planning, smart manufacturing

## Abstract

In the context of smart manufacturing, improving the quality and efficiency of process planning, especially in the processing of complex parts, has become a key factor influencing the level of intelligence in manufacturing systems. However, most current process planning methods still heavily rely on manual expertise, leading to problems such as difficulty in knowledge reuse, low planning efficiency, and slow response times, which are inadequate to meet the diverse and changing needs of engineering applications. To address these issues, this paper proposes an algorithm for Assembly Process Reasoning and Decision-making based on Bidirectional Long Short-Term Memory with Attention (AP-BiLSTM-ATT), which aims to deeply explore the hidden relationships between the multi-dimensional features of parts and process plans, thereby achieving probabilistic modeling of process decisions. Specifically, the attributes, geometric features, and historical process plans of parts are first labeled and vectorized, transforming traditional process knowledge into structured data representations suitable for deep learning models. A BiLSTM network model, integrated with a multi-head attention mechanism, is then constructed to capture contextual dependencies and semantic weight distributions between features, enhancing the model’s ability to express complex process relationships. During training, the model learns the mapping distribution between features and processes from a large-scale historical process dataset, enabling intelligent reasoning and recommendation of process plans for new parts. The results show that this method outperforms traditional methods in terms of accuracy, response speed, and generalization ability in process planning, providing effective support for enhancing the intelligence of complex part process planning and laying a foundation for the structured expression and intelligent application of manufacturing process knowledge.

## 1 Introduction

With the continuous advancement of intelligent manufacturing technology and the deep integration of advanced manufacturing and information technologies, manufacturing enterprises are achieving key breakthroughs in improving product quality, increasing production efficiency, and reducing production costs. Intelligent manufacturing has widely penetrated all stages of the product lifecycle, including product design, production manufacturing, and service maintenance. Research shows that as emerging manufacturing industries accelerate the deployment of intelligent technologies, the production efficiency of their manufacturing systems has increased by 17%–20%. However, as a critical link between product design and production execution, manufacturing process planning still relies primarily on human-machine interaction, with its core decision-making process heavily dependent on engineers’ professional knowledge and experience.

**Figure 1:**
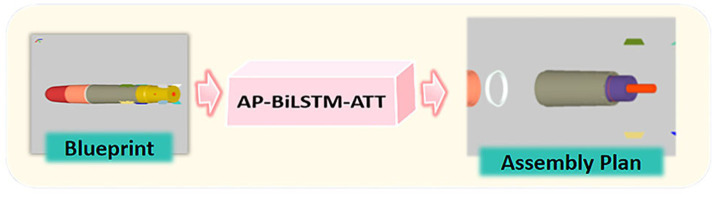
Simulation results for the network.

In this context, the role of process planning in ensuring product quality, improving machining efficiency, and optimizing production costs becomes particularly significant. Especially in the manufacturing of complex structural parts, efficiently reasoning out the most appropriate process plan from the multi-dimensional features of parts has always been a core challenge of intelligent process planning. In contrast, traditional process planning often requires engineers to manually devise plans based on their experience, which is not only time-consuming and inefficient but also prone to subjective influences, making it difficult to meet the modern manufacturing industry’s demands for rapid response and precise decision-making. The conceptual flow of this planning task is depicted in Figure 1.

With the gradual popularization of modern Computer-Aided Process Planning (CAPP) systems, enterprises have accumulated a wealth of historical process data. How to effectively extract valuable knowledge from this large amount of historical data and utilize it through intelligent methods has become a core issue in process reasoning and decision support. For example, Agrawal et al. (2009) proposed a multi-agent distributed CAPP system composed of a global management agent, design agent, and optimization agent. Using a backward-chaining reasoning mechanism, the system realized intelligent decision-making for complex processes. Qian et al. (2023) built a process-oriented knowledge ontology library for assembly sequence planning and applied a Mixed-Integer Linear Programming (MILP) model to optimize assembly actions and part sequences with the objective of minimizing assembly time. Combined with a human-computer collaboration visualization tool, they achieved rapid automatic generation of assembly plans. Mou and Gao (2020) proposed a fuzzy comprehensive evaluation method based on historical machining data for assessing process plan reliability, improving robustness in multi-objective and uncertain scenarios. Wang et al. (2015) modeled the process planning problem as a directed graph and used a two-stage ant colony algorithm for parallel optimal path search, significantly reducing production costs and improving algorithm efficiency. In addition, Rojek (2010) evaluated the performance of Multilayer Perceptrons (MLP) and Radial Basis Function (RBF) networks in intelligent CAPP systems, demonstrating that they outperform traditional rule-based methods in tool selection and operation sequencing. Deb et al. (2006) proposed a neural network-based method for selecting machining operations, automatically determining process parameters and tool configurations for rotationally symmetric parts.

However, traditional methods still face several critical challenges: high costs for knowledge acquisition and maintenance, with rules, ontologies, and templates requiring frequent updates by experts; insufficient structuring of historical data, making it difficult to fully leverage heterogeneous and multi-source process instances; and limited real-time responsiveness to dynamic environments, hindering rapid adjustments to production changes.

To overcome these bottlenecks, researchers have recently introduced machine learning, deep learning, and reinforcement learning technologies into CAPP systems. Zhang et al. (2022) developed an intelligent decision-making system that maps assembly units and process features into a multidimensional vector space, optimizing assembly sequence planning via supervised learning models, thereby significantly enhancing the system’s generalization and automation capabilities. Jiang et al. (2024) proposed a fine-grained assembly sequence planning method based on knowledge graphs and deep reinforcement learning, where assembly operations are modeled as continuous and discrete processes, constructing a dynamic graph and applying an improved Deep Q-Network (DQN) to enable real-time decision-making under complex constraints with hierarchical Seq2Seq neural reasoning. Zhu et al. (2024) designed a two-stage Seq2Seq neural network that captures both assembly sequences and contact point selections through hierarchical reasoning, providing highly flexible process planning for robotic assembly. Mortlock et al. (2021) integrated Graph Neural Networks (GNNs) within a cognitive digital twin framework to couple real-time shop floor data with process models, supporting dynamic re-planning and predictive maintenance.

Despite the significant progress made by current intelligent process reasoning methods in improving decision-making efficiency and accuracy, they still face several major challenges. First, deep learning methods heavily rely on large amounts of accurately labeled historical data, and acquiring high-quality labeled data is both time-consuming and costly for manufacturing enterprises. Meanwhile, although knowledge graphs effectively integrate process knowledge, their construction and maintenance are complex and labor-intensive, becoming increasingly difficult as the system evolves.

To address these challenges, this paper proposes the following contributions:

A process reasoning model (AP-BiLSTM-ATT) based on part attributes, geometric features, and process plan labeling and vectorization is proposed. The model outputs a probability distribution over candidate process plans via a final softmax layer, enabling uncertainty quantification and ranking of multiple feasible plans by their likelihood. This model effectively captures the multidimensional features of parts, reduces reliance on large-scale labeled datasets, and provides efficient process recommendations.

An attention mechanism (ATT) is introduced into the process reasoning framework, enabling the model to dynamically focus on key information in part features. This enhances the precision of process plan reasoning, reduces the dependency on complex ontology and knowledge graph construction, and improves model interpretability.

Experimental results show that the proposed method can quickly and accurately recommend optimal process plans for new parts without relying on complex graphs or massive labeled data. The results demonstrate that the method significantly outperforms traditional approaches in terms of accuracy, recommendation speed, and interpretability, effectively improving process planning efficiency.

## 2 Related work

Assembly Process Planning (APP), as a key stage within the smart manufacturing workflow, aims to generate efficient and rational assembly plans while satisfying assembly constraints and resource limitations. Among its components, Assembly Sequence Planning (ASP) constitutes the core of assembly, directly impacting the efficiency, quality, and production cost of product assembly. Consequently, how to efficiently and accurately achieve the automatic generation of assembly sequences has become a major focus of research both domestically and internationally.

### 2.1 Traditional rule-based and heuristic search methods

Early research primarily relied on traditional methods based on geometric features and assembly constraint rules. Torres et al. (2003) proposed an assembly relation model and solved the assembly sequence planning problem by leveraging the reverse logic of disassembly and assembly. Dini et al. (1999) developed a mathematical representation model of the assembly process based on the assembly interference matrix and contact matrix, achieving the quantitative evaluation and selection of assembly sequences. Although these methods offer good intuitiveness and interpretability, their modeling efficiency and degree of automation remain limited when applied to industrial scenarios characterized by increasingly complex assembly structures and a large number of components.

To overcome these bottlenecks, a significant body of research in recent years has introduced heuristic and intelligent optimization algorithms to enhance the efficiency of assembly sequence planning. For instance, Chen and Liu (2001) proposed an adaptive genetic algorithm (GA) to address the poor adaptability of traditional GA operators. Abdullah et al. (2019) constructed multi-objective assembly optimization models using the Artificial Bee Colony algorithm and the Moth-Flame Optimization algorithm, respectively. Beyond these, variants of Particle Swarm Optimization (PSO) have been explored. Zhang (2023) developed an Improved PSO (IPSO) that redefines particle update rules and incorporates GA-style mutation to accelerate convergence and escape local optima. Wu et al. (2019) applied a PSO-based method leveraging assembly direction, interference, and sequence-relation matrices to obtain optimal sequences under fixture constraints. To generate diverse Pareto-optimal assembly plans, Wan et al. (2024) introduced a Multiple Optimal Solutions GA (MOSGA), balancing assembly time and resource consumption for large modular assemblies MDPI. Hybrid swarm–behavior algorithms have also been developed. Wu et al. (2019) proposed SOS-ACO, coupling Symbiotic Organisms Search with Ant Colony Optimization to adaptively tune pheromone parameters, achieving near-optimal sequences in fewer iterations and Zhang et al. (2025) presented an SOS-PSO hybrid that integrates immune-inspired selection with PSO, demonstrating superior robustness and convergence in constrained multi-agent assembly scenarios.

Although these methods have demonstrated promising potential in improving assembly efficiency and reducing resource consumption, they fundamentally remain heuristic search frameworks—sensitive to initial parameter settings and prone to local optima in large-scale combinatorial spaces, with limited capabilities for deep modeling of assembly knowledge.

### 2.2 Machine learning and deep learning methods

In order to further enhance the intelligence level of assembly process planning, some studies have begun exploring the application of machine learning methods. Research has been conducted to develop an assembly prediction system based on artificial neural networks. This system constructs an assembly evaluation function and employs supervised learning to predict and optimize assembly steps. Furthermore, a hybrid assembly sequence optimization model has been proposed, which integrates multiple neural network structures with K-means clustering. Although these approaches perform well in specific experimental scenarios, their relatively shallow network structures and limited capability to model temporal features restrict their ability to fully capture the contextual dependencies and long-term constraint information inherent in the assembly process. Guo et al. (2024) proposed a DRL method with multiple starting-node exploration was introduced to address dynamic changes in machining resources. By augmenting the state-space exploration with varied initial conditions, it achieved superior resource utilization and planning robustness compared to standard RL baselines. Li et al. (2024) has been modeled as a Markov Decision Process and solved via a heterogeneous Graph Neural Network combined with Proximal Policy Optimization. This end-to-end approach captured operation–machine relationships and outperformed MILP-based methods in both solution quality and computation time on large-scale instances. In assembly sequence planning, Neves and Neto (2022) applied DRL with parametric action spaces and dual reward signals—reflecting user ergonomic preferences and cycle-time minimization—comparing A2C, DQN, and Rainbow; Rainbow achieved near-optimal performance after 10,000 episodes, surpassing tabular Q-Learning in complex deterministic and stochastic scenarios. For additive manufacturing, Mozaffar et al. (2020) developed a DRL-based toolpath planning platform that learns deposition strategies under dense reward structures, demonstrating high fidelity to expert-designed toolpaths and adaptability to arbitrary geometries. Wang et al. (2023) proposed a dual-attention DRL model was proposed for flexible job shop scheduling—a close relative of process planning—where interconnected operation-message and machine-message attention blocks guide priority decisions. This framework achieved solution quality comparable to exact methods on benchmark tasks, highlighting the promise of attention architectures in capturing complex process-machine interactions.

It is worth noting that deep learning-based methods for assembly sequence modeling are still in an exploratory stage, and related research remains relatively scarce. In tackling complex assembly tasks characterized by sequentiality and structural dependency, sequence modeling capability becomes critical. The selection of BiLSTM with attention is theoretically grounded in the sequential nature and long-range dependencies inherent in process planning tasks. Process plans typically involve ordered sequences of operations where previous decisions influence subsequent steps, and critical dependencies may span across multiple operations. BiLSTM effectively captures bidirectional contextual dependencies in these process sequences, while the attention mechanism dynamically weights important features and operations, addressing both local patterns and long-range dependencies that are characteristic of complex manufacturing processes. Bidirectional Long Short-Term Memory (BiLSTM) networks, known for their ability to capture both historical and future information simultaneously, have been widely used in fields such as semantic recognition and event prediction. When combined with the attention mechanism, the network can dynamically allocate focus weights, emphasizing key steps in the assembly process, thereby enhancing the model’s ability to understand complex assembly logic.

## 3 Materials and methods

**Figure 2:**
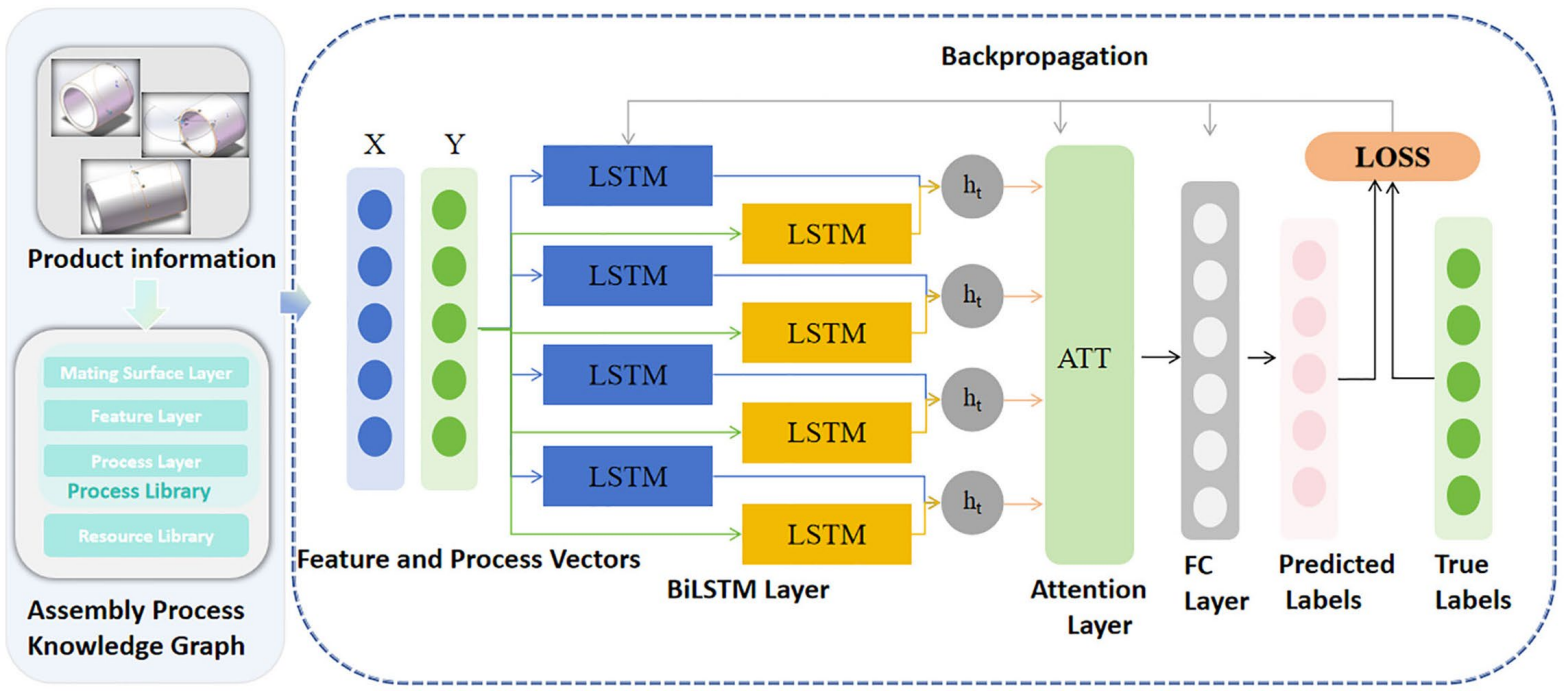
The proposed algorithm: AP-BiLSTM-ATT.

In this paper, we first extract critical assembly information from the three-dimensional (3D) CAD models of products, including the mating surfaces of each part, geometric attributes, and inter-surface constraints within the assembly. Based on the extracted information, we construct an assembly feature representation model oriented toward process planning, thereby establishing a training sample mapping between assembly features and typical process operations. This mapping captures implicit rules linking part assembly types, mating attributes, and corresponding assembly operations—such as insertion, press-fitting, welding, screwing, and positioning/clamping—which serve as supervised labels for the subsequent deep learning model training. The overall architecture of the proposed AP-BiLSTM-ATT framework is illustrated in Figure 2.

### 3.1 Data preprocessing

The core objective of the AP-BiLSTM-ATT algorithm is to maximize the prediction probability of part process plans based on the key attributes and features of the parts during model training. To achieve this goal, it is essential to first prepare a training dataset that includes the mapping relationships between parts and their corresponding process plans. During the training phase, the system learns the nonlinear mapping between part features and process plans, thereby enabling efficient and accurate process recommendations.

The data preprocessing involves a detailed “Labeling and Vectorization” procedure to transform part attributes and process knowledge into a structured format. First, process plan labeling is performed: each unique sequence of machining operations (e.g., “Drilling → Rough Turning → Finish Turning”) is assigned a unique categorical label, which serves as the target for the model’s multi-class prediction task. Second, part feature vectorization is conducted: categorical features (e.g., material types) are encoded using one-hot encoding; numerical features (e.g., diameter, length) are normalized to a [0, 1] range; and machining operations are mapped to dense embedding vectors through a trainable embedding layer, enabling the model to learn semantic relationships between operations.

In the specific data preprocessing phase, the training set consists of A part samples and their corresponding B process plans, forming a large number of part–process plan pairs as training examples. Each training sample not only contains the basic coding information of the part (such as part ID and type) but also includes the code of the associated process plan and multi-dimensional feature information of the part (such as dimensional parameters, surface requirements, material types, and structural complexity). These inputs are transformed into feature vectors that feed into the model to establish a deep mapping relationship between part features and process operations.

To enhance data quality and model performance, additional preprocessing steps are employed. Encoding and vectorization ensure all features are in neural-network-readable format, while data augmentation through slight feature value perturbation expands the effective sample size and improves model robustness to input variations. Collectively, these steps ensure the input data maintains its integrity while being optimally prepared for the AP-BiLSTM-ATT model, establishing a solid foundation for reliable process planning recommendations.

### 3.2 AP-BiLSTM-ATT

**Figure 3:**
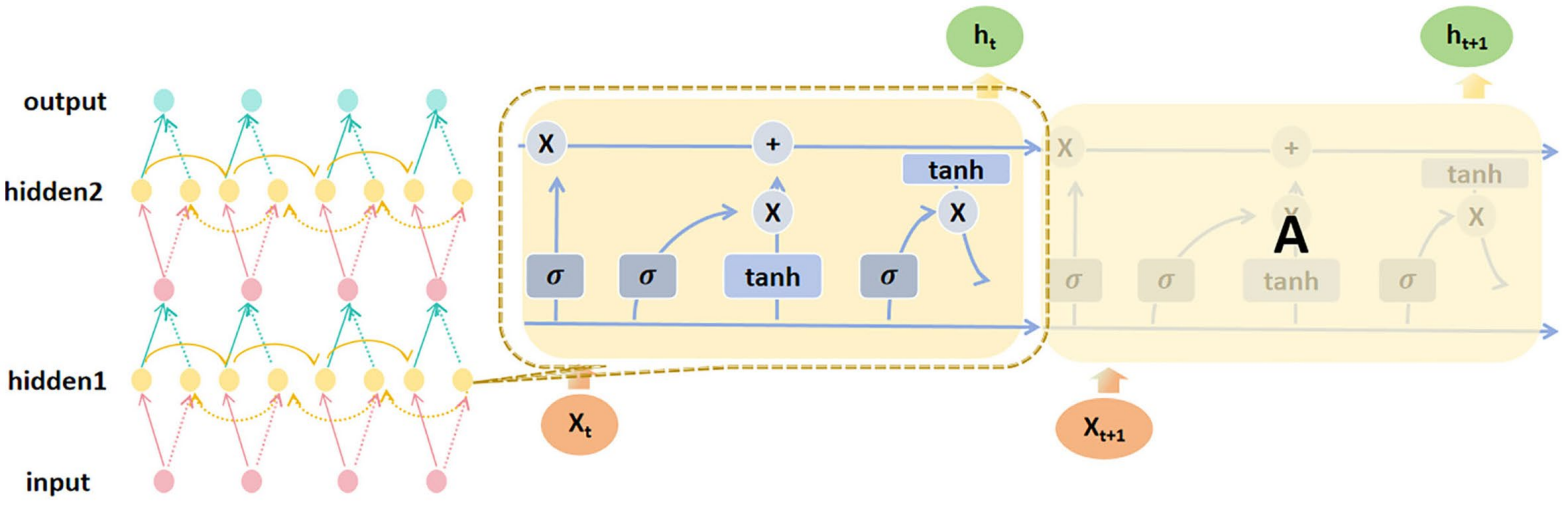
BiLSTM architecture diagram.

**Figure 4:**
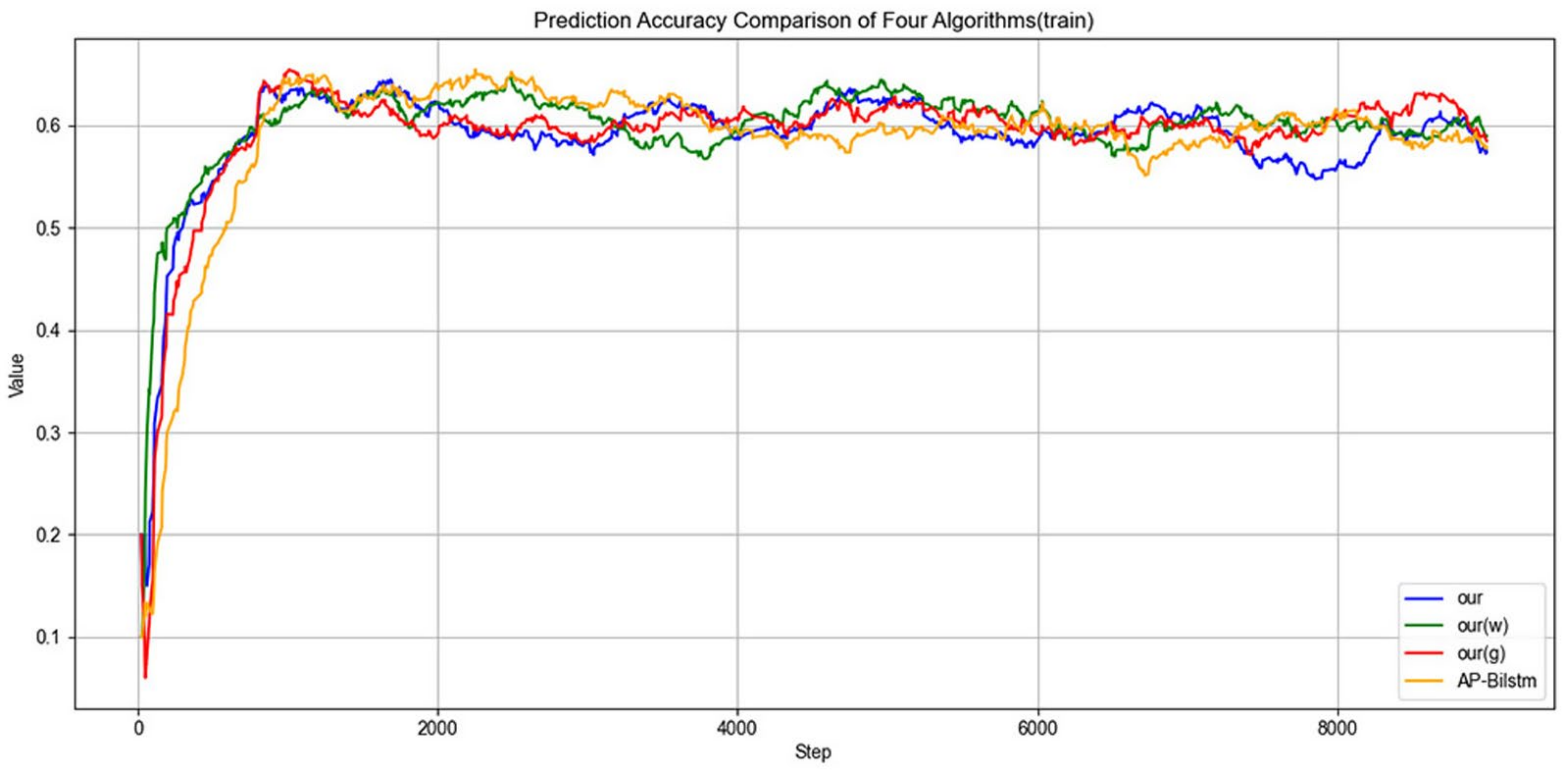
Attention mechanism architecture diagram.

Specifically, this study incorporates Bidirectional Long Short-Term Memory (BiLSTM) networks combined with Attention Mechanism to address the problem of process plan prediction. BiLSTM is a deep learning model capable of capturing both forward and backward dependencies within sequential data, making it particularly suitable for handling complex sequential data and long-range dependencies. The detailed structure of the BiLSTM component is depicted in Figure 3. The architectural selection of BiLSTM with attention is strategically aligned with the fundamental characteristics of process planning tasks. In manufacturing environments, process sequences exhibit strong temporal dependencies where early-stage decisions (e.g., material selection and rough machining parameters) fundamentally constrain subsequent operations (e.g., finishing and quality control). The BiLSTM component excels at modeling these bidirectional process flows, while the attention mechanism, whose architecture is detailed in Figure 4, addresses the critical challenge of long-range dependencies in manufacturing processes. This synergistic combination allows the model to not only understand local sequential patterns but also recognize global process constraints that span multiple manufacturing stages. By leveraging its bidirectional structure, BiLSTM performs computations in both the forward and backward directions of a time sequence, enabling a more comprehensive understanding of the contextual information in the input data. This significantly enhances the model’s ability to extract and model features. At each time step *t*, the forward LSTM sequentially computes the current hidden state ${h_{t}}^{\left(f\right)}$ and cell state $c_{t}^{\left(f\right)}$ using the following steps.

First, the Input Gate determines the influence of the current input $x_{t}$ on the cell state. The output $i_{t}$ of the input gate is calculated as shown in Equation 1:


(1) $$i_{t}=\sigma (W_{i}x_{t}+U_{i}h_{t-1}^{\left(f\right)}+b_{i})$$

where $W_{i}$ is the input weight matrix, $U_{i}$ is the hidden state weight matrix, $b_{i}$ is the bias term, and σ is the sigmoid activation function. $h_{t-1}^{\left(f\right)}$ is the hidden state from the previous time step.

Next, the Forget Gate controls the proportion of information from the previous time step’s cell state $c_{t-1}^{\left(f\right)}$ to be retained in the current cell state $c_{t}^{\left(f\right)}$ . The output $f_{t}$ of the forget gate is computed as shown in Equation 2:


(2) $$f_{t}=\sigma (W_{f}x_{t}+U_{f}h_{t-1}^{\left(f\right)}+b_{f})$$

where $W_{f}$ and $U_{f}$ are the forget gate weight matrices, and $b_{f}$ is the bias term.

Then, the Output Gate determines the current hidden state $h_{t}^{\left(f\right)}$ . The output $o_{t}$ of the output gate is calculated as shown in Equation 3:


(3) $$o_{t}=\sigma (W_{o}x_{t}+U_{o}h_{t-1}^{\left(f\right)}+b_{o})$$

where $\;W_{o}$ and $U_{o}$ are the output gate weight matrices, and $b_{o}$ is the bias term.

Next, the Cell State is computed by combining the previous time step’s cell state and the contributions from the input and forget gates. The update equation for the cell state is as shown in Equation 4:


(4) $$c_{t}^{\left(f\right)}=f_{t}\cdot c_{t-1}^{\left(f\right)}+i_{t}\cdot \tanh(W_{c}x_{t}+U_{c}h_{t-1}^{\left(f\right)}+b_{c})$$

where $W_{c}$ and $U_{c}$ are the memory update weight matrices, and $b_{c}$ is the bias term. The tanh is the hyperbolic tangent activation function. This equation combines the influence of the forget gate, which retains the previous memory information, and the input gate, which introduces new information from the current time step to form the updated cell state $c_{t}^{\left(f\right)}$ .

Finally, the hidden state is computed as the non-linear combination of the output gate and the current cell state, as shown in Equation 5:


(5) $$h_{t}^{\left(f\right)}=o_{t}\cdot \tanh(c_{t}^{\left(f\right)})$$

Through these steps, the forward LSTM effectively captures the long-term dependencies in the input sequence. At each time step, the forward LSTM updates its hidden state $h_{t}^{\left(f\right)}$ and cell state $c_{t}^{\left(f\right)}$ , which are then passed on to the next time step.

Similarly, the backward LSTM processes the input features starting from the end of the sequence. It computes the hidden state and cell state in the reverse order. The forward and backward LSTM hidden states are then concatenated to form the final output of the bidirectional LSTM, which fully utilizes both past and future information from the input sequence.

After obtaining the concatenated bidirectional hidden representations from the BiLSTM network, an attention mechanism is introduced to further enhance the model’s ability to capture critical information. While BiLSTM can effectively model long-range dependencies and contextual relationships in the input sequence, its output treats all time steps equally. This uniform treatment may dilute the influence of key time-step features on the final representation. The attention mechanism addresses this limitation by allowing the model to learn the relative importance of each time step in the sequence, enabling it to focus on features most relevant to process plan prediction.

Formally, let the hidden state sequence output by BiLSTM be $H=\{h_{1},h_{2},\ldots ,h_{T}\}$ , where $h_{t}\in {\mathbb{R}}^{d}$ denotes the hidden state at time step *t*. The attention mechanism first computes a relevance score $e_{t}$ for each hidden state, as shown in Equation 6:


(6) $$e_{t}=v^{⊤}\tanh(W_{a}h_{t}+b_{a})$$

where $W_{a}\in {\mathbb{R}}^{d_{a}\times d}$ is a learnable weight matrix, $v\in {\mathbb{R}}^{d_{a}}$ is a weight vector, and $b_{a}\in {\mathbb{R}}^{d_{a}}$ is a bias term. These scores are then normalized by a softmax function to obtain the attention weights $\alpha_{t}$ , as shown in Equation 7:


(7) $$\alpha_{t}=\frac{\exp(e_{t})}{\sum_{i=1}^{T}\exp(e_{i})}$$

The context vector **c**, which serves as a weighted representation of the sequence, is computed as shown in Equation 8:


(8) $$c=\sum_{t=1}^{T}\alpha_{t}h_{t}$$

This context vector integrates information from all time steps, with higher weights assigned to more informative steps, thereby enhancing the model’s representation of critical process-related features.

The multi-head attention mechanism extends the standard attention by employing multiple attention heads (*h* = 8) in parallel. Each head learns distinct feature representations from different subspaces, enabling the model to capture diverse aspects of process planning semantics. Formally, for head i, the attention output is computed as shown in Equation 9:


(9) $$\mathrm{hea}d_{i}=\text{Attention}(HW_{i}^{Q},HW_{i}^{K},HW_{i}^{V})$$

where $W_{i}^{Q}\in R^{d\times d_{k}},W_{i}^{K}\in R^{d\times d_{k}}$ , and $W_{i}^{V}\in R^{d\times d_{v}}$ are learnable projection matrices for queries, keys, and values respectively, with $d_{k}=d_{v}=d/h=12$ . The outputs of all heads are concatenated and then linearly transformed, as shown in Equation 10:


(10) $$\text{MultiHead}(H)=\text{Concat}(h\mathrm{ea}d_{1},,..,,h\mathrm{ea}d_{h})W^{O}$$

where $W^{O}\in R^{h\cdot d_{v}\times d}$ is the output projection matrix. This architectural design allows the model to jointly attend to information from different representation subspaces, effectively capturing various feature interactions in process planning, such as the relationships between geometric features, material properties, and process parameters.

A key innovation of our framework is its probabilistic output, which transforms the model from a deterministic classifier into a decision-support tool. Subsequently, the context vector **c** is passed through a fully connected layer followed by a softmax classifier to generate the predicted probability distribution over the candidate process plan labels, as shown in Equation 11:


(11) $$\hat{y}=\text{Softmax}(W_{s}c+b_{s})$$

Here, $W_{s}\in {\mathbb{R}}^{K\times d}$ and $b_{s}\in {\mathbb{R}}^{K}$ are the weights and bias of the classification layer, and *K* is the number of process plan categories. $\hat{y}$ represents a probability distribution where each element denotes the likelihood of a corresponding process plan being the optimal choice. This output allows for: (1) Quantifying the uncertainty of the top recommendation, and (2) Ranking and presenting multiple feasible alternative plans to the process planner, thereby enabling more informed and flexible decision-making in a smart manufacturing context.

The entire model is trained with a cross-entropy loss function. This function quantifies the discrepancy between the predicted probabilities $\hat{y}\;$ and the ground truth labels **y**, as presented in Equation 12:


(12) $$ℒ=-\sum_{i=1}^{K}y_{i}\log({\hat{y}}_{i})$$

where $y\in {\left\{0,1\right\}}^{K}$ is a one-hot encoded vector representing the true label, and ${\hat{y}}_{i}$ denotes the predicted probability for class *i*. This loss is minimized during training using backpropagation to update all model parameters. The complete training procedure outlined above is summarized in Algorithm 1.

ALGORITHM 1 Framework of AP-BiLSTM-ATT for process planning.
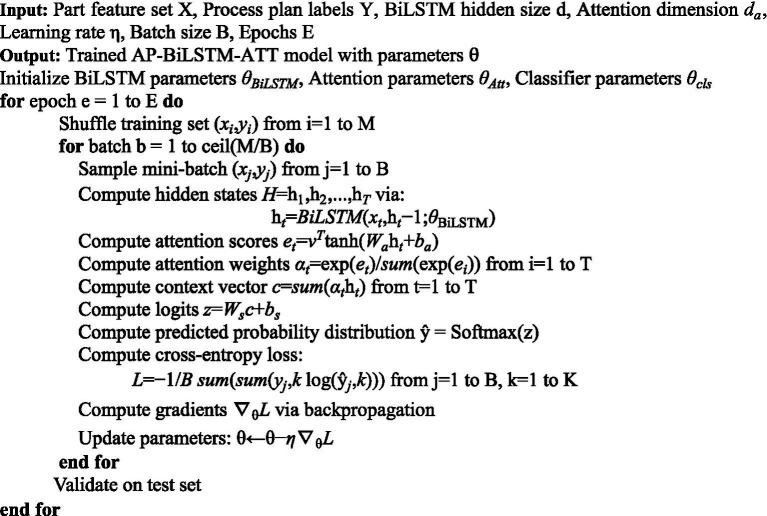

## 4 Experiments

### 4.1 Dataset

The custom dataset employed in this study consists of 1,000 instances of machining process data for various parts. This dataset was synthetically constructed to support research in data-driven process planning, comprising typical precision components such as shafts, plates, and housings to ensure diversity in part geometry and function. The historical process plans were generated based on domain expertise and standard manufacturing guidelines, with each plan representing a feasible sequence of operations (e.g., “Drilling → Rough Turning → Finish Turning”). These sequences were subsequently validated through simulation to ensure logical consistency and adherence to machining principles. Each instance includes a part description—encompassing material type (e.g., copper, 304 stainless steel, 45 steel, aluminum), geometric attributes (end face type, diameter, length), and technical specifications (flatness tolerance, surface roughness, hardness)—and its corresponding machining process plan, which records the sequential operations from rough to finish machining. This dataset provides a foundation for developing and validating intelligent process recommendation systems. Each instance is composed of two key components: part description and machining process plan. The part description includes information such as material type (e.g., copper, 304 stainless steel, 45 steel, aluminum), end face type, flatness tolerance, part diameter, part length, hole requirements, coating requirements, surface roughness, and hardness grade. The machining process plan records the sequence of operations performed on each part, encompassing various stages from rough to finish machining, such as drilling, rough turning, finish turning, boring, coating, tapping, cutting, and surface treatment. This dataset reflects the diversity of materials and machining processes, providing valuable support for machine learning-based machining process recommendation systems. It aims to facilitate automated prediction and optimization, improving production efficiency while reducing manual intervention.

### 4.2 Experimental setup

The experiments in this study were conducted on an Intel(R) Core(TM) i7-8550 U CPU and an NVIDIA GeForce RTX 3080 Ti GPU with 8GB of RAM. The experimental model is implemented using the TensorFlow framework and employs a Bidirectional Long Short-Term Memory (Bi-LSTM) network coupled with an Attention Mechanism. Specifically, the Bi-LSTM consists of forward and backward LSTM units, each containing 100 hidden neurons and utilizing the ReLU activation function. Dropout regularization is applied, with a dropout keep probability of 0.7 for both the embedding and RNN layers. The Attention Mechanism weights the outputs of the LSTM layers to enhance the model’s focus on critical information. Other hyperparameters, including embedding layer dimensions and L2 regularization, are detailed in Table 1. During training, the batch size is set to 160, the learning rate is initialized to 0.001, and the Adam optimizer with a learning rate decay strategy is employed. The dataset is divided into a 90 training set and a 10 validation set, with cross-validation used to ensure the robustness of the experiment.

**Table 1:** The description of experimental parameters.

Parameter	Configuration
System	Windows
GPU	GeForce RTX 3080 Ti
Coding environment	Python3.7
Tensorflow	v2.3.0
Numpy	v1.19.5
Rnn-dropout-keep-prob	0.7
Dropout-keep-prob	0.5
Emb-dropout-keep-prob	0.7
Embedding-dim	100
L2-reg-lambda	1e-5
Batch-size	10
Num-epochs	100
Evaluate-every	100
Learning rate	0.001
Decay-rate	0.9
Hidden-size	100
Dev-sample-percentage	0.1

To thoroughly evaluate the performance of the proposed AP-BiLSTM-ATT model (denoted as Ours), we compare it against several carefully designed baseline and ablation models to isolate the contribution of key components.

AP-BiLSTM: This is an ablation model that removes the attention mechanism from our full model. It retains the same BiLSTM layers but uses the last hidden state for classification instead of the attention-weighted context vector. The comparison between Ours and AP-BiLSTM is designed to directly quantify the performance gain attributable to the attention mechanism.

Ours(g) & Ours(w): These two model variants were designed to evaluate the impact of different word embedding initialization strategies on performance. Specifically, “Ours(g)” initializes the embedding layer using pre-trained GloVe vectors, while “Ours(w)” initializes it with vectors generated by the Word2Vec method. Our final model (Ours) employs an end-to-end trained embedding layer with random initialization. Their inclusion aims to demonstrate that our final choice of an end-to-end training strategy outperforms approaches reliant on external pre-trained models, thereby highlighting the simplicity and effectiveness of our final architecture.

### 4.3 Experimental evaluation metrics

This work uses five key metrics to evaluate the performance of the process plan prediction model: HR@n, MRR@n, Process Plan Prediction Accuracy (Seq-Acc), Computation Time (CT), and Loss. HR@n (Hit Rate at Top n) measures whether the predicted process plan is ranked within the top n positions in the candidate list. If the predicted result is within the top n, the value is 1; otherwise, it is 0. MRR@n (Mean Reciprocal Rank at Top n) calculates the average reciprocal rank of the first correct prediction in the candidate list, where the value at the k-th position is 1/k, and if the prediction is not in the candidate list, the value is 0. Process Plan Prediction Accuracy (Seq-Acc) evaluates whether the model successfully predicts the correctness of the entire process plan, indicating the model’s ability to match the process plan. Computation Time (CT) measures the time required for the model to make the process plan prediction, reflecting the model’s efficiency, which is especially significant in practical applications. Loss represents the difference between the model’s output and the true labels; a lower loss indicates better model performance. By utilizing these metrics, the accuracy, efficiency, and optimization of the model can be comprehensively assessed.

### 4.4 Experimental result analysis

The experiment focuses on analyzing the model’s performance on both the training and validation sets, with particular attention to the changes in accuracy and loss.

**Figure 5:**
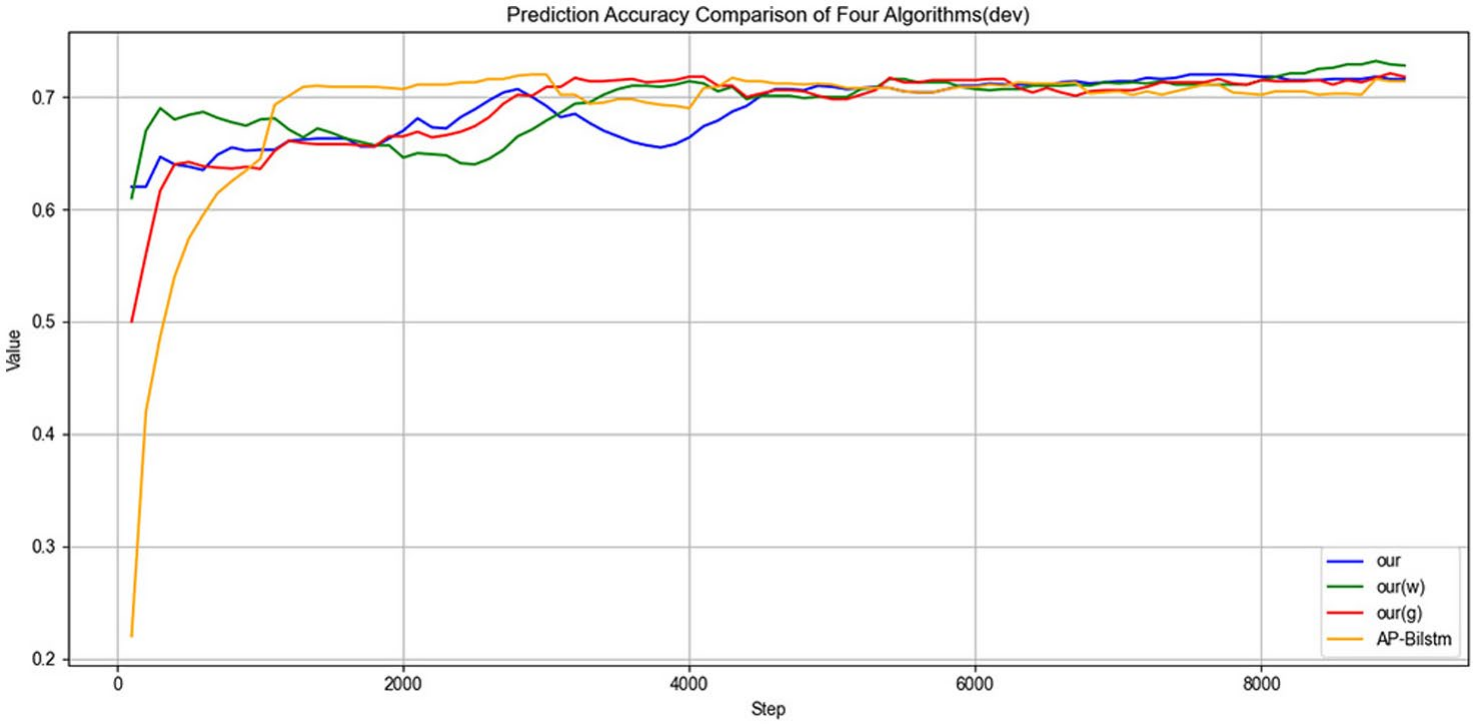
Training accuracy over epochs.

(1) Accuracy: First, Figure 5 illustrates the variation in accuracy on the training set. As the training progresses, the model’s accuracy on the training set gradually increases, indicating that the model successfully learns the features of the data and optimizing its internal parameters to enhance prediction capability. Although there may be some fluctuations in the early stages, the overall accuracy stabilizes and steadily rises, reflecting the continuous improvement of the training process.

**Figure 6:**
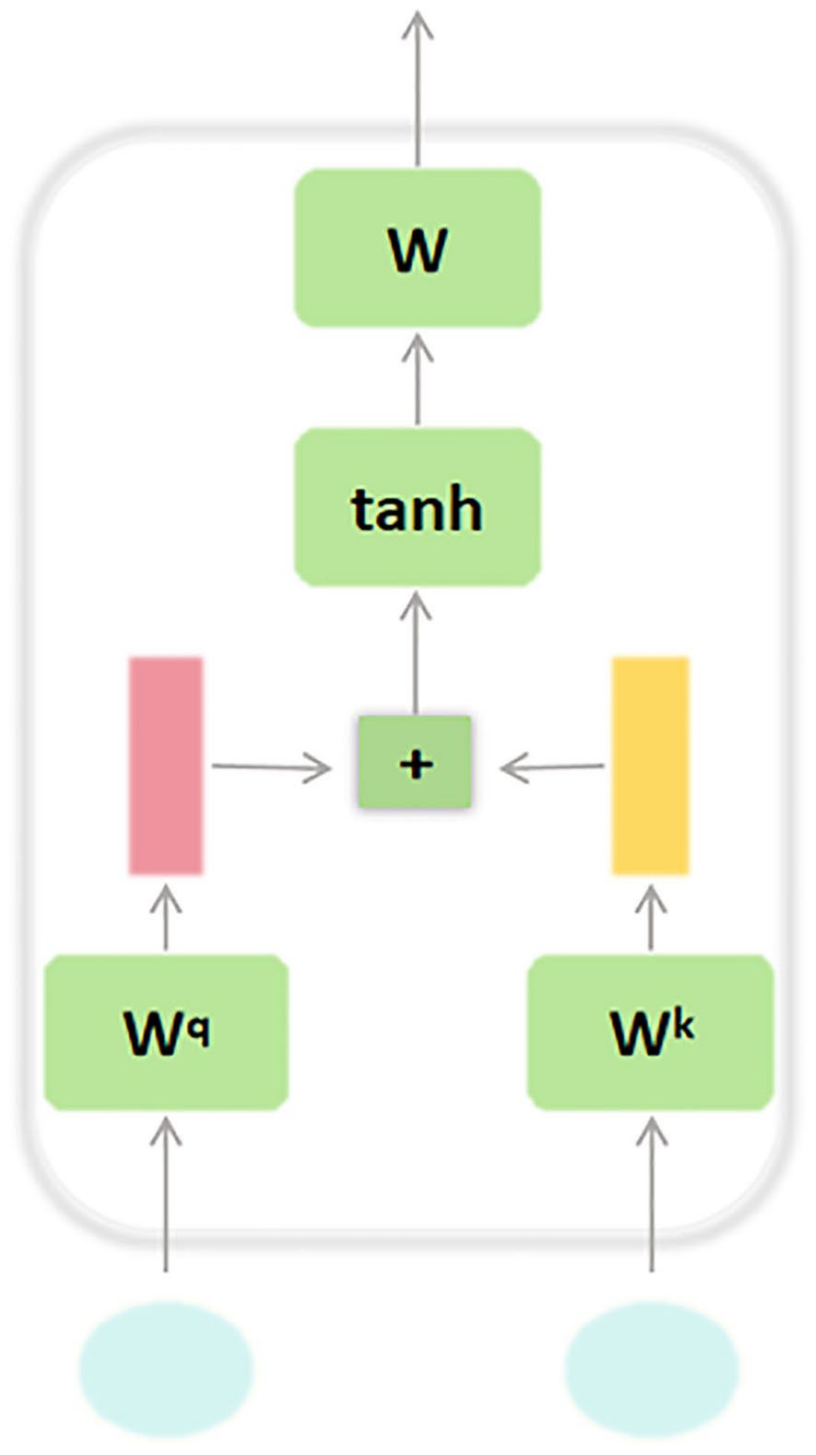
Validation accuracy over epochs.

Figure 6 illustrates the accuracy trends on the validation set. It can be observed that the proposed method achieves a higher initial accuracy compared to other algorithms during the validation process, indicating that the model possesses strong feature extraction capabilities at an early stage. As training progresses, the proposed method consistently maintains its advantage in accuracy and ultimately converges to a higher accuracy level than the competing algorithms. These results demonstrate that the proposed model not only exhibits strong learning ability in the early training phase but also shows greater stability and convergence performance throughout the training process, thereby confirming its superiority in terms of generalization capability and predictive effectiveness.

**Figure 7:**
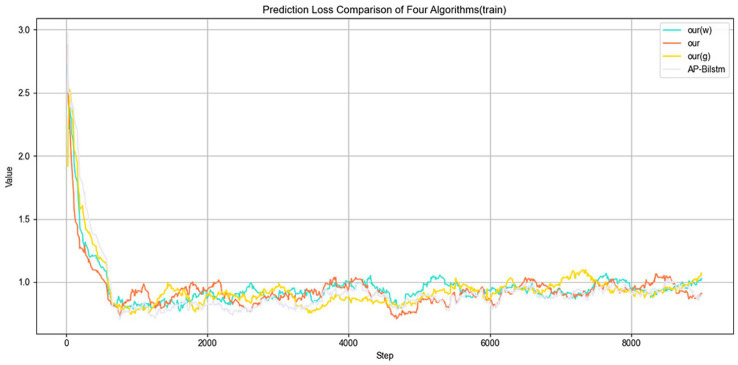
Training loss over epochs.

(2) Loss: Figure 7 illustrates the variation in Loss on the training set. As training progresses, the Loss gradually decreases, indicating that the model is reducing prediction errors during the optimization process. Although some fluctuations may occur in the early stages, the Loss stabilizes and converges to a lower level as training continues, suggesting that the model progressively improves its fit to the training data.

**Figure 8:**
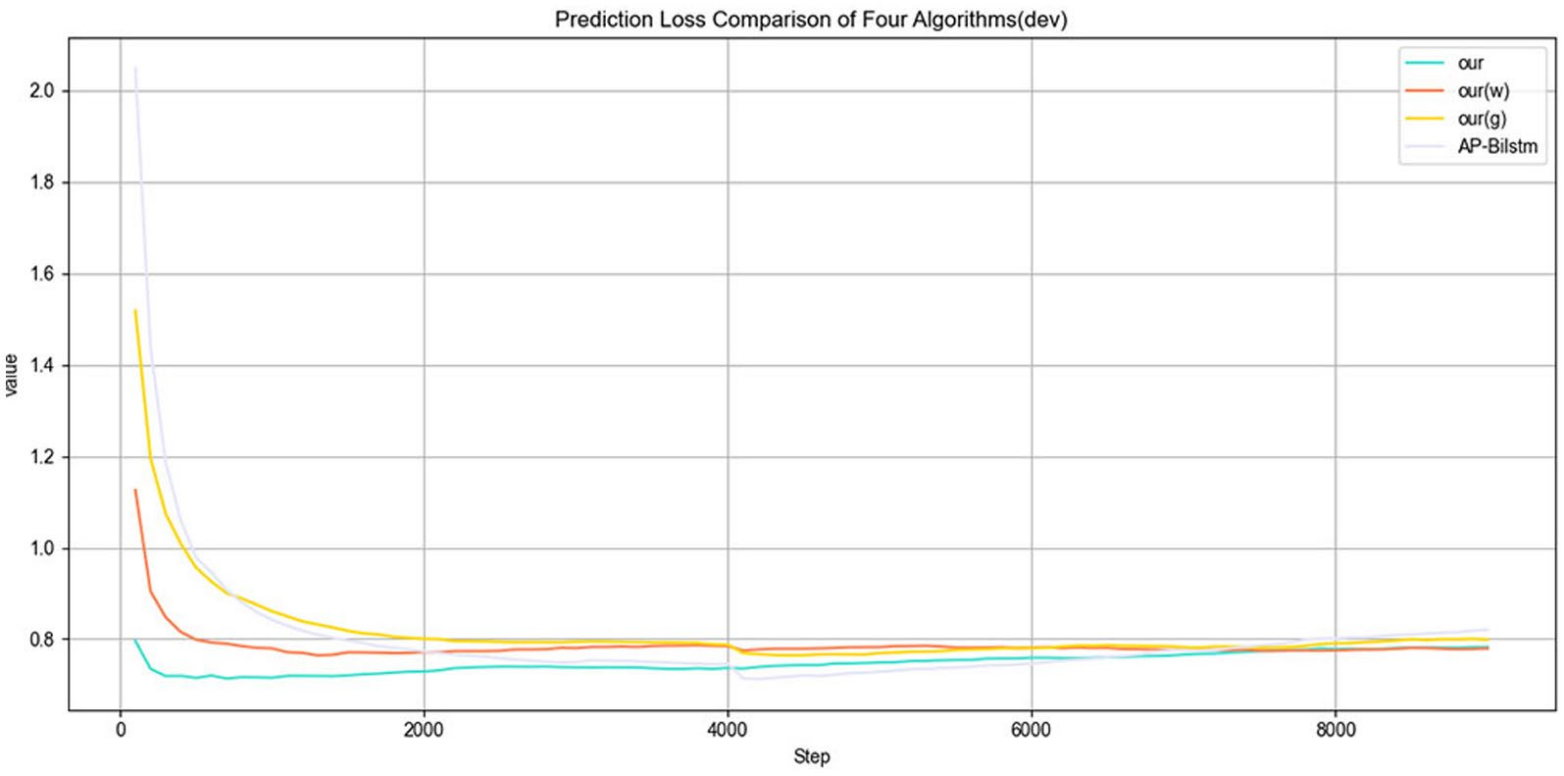
Validation loss over epochs.

Figure 8 illustrates the variation in Loss on the validation set. It is evident that the proposed method consistently achieves lower Loss values throughout the validation process compared to other algorithms. This indicates that the model is more effective in minimizing prediction errors on unseen data, demonstrating superior generalization capability. Moreover, the lower validation Loss suggests that the model maintains a good fit without overfitting, thereby exhibiting enhanced robustness and stability.

(3) HR@k and MRR@k: Based on the evaluation metrics presented in Table 2, we can observe the model’s performance in recommendation accuracy at various positions.

**Table 2:** HR@k and MRR@k evaluation metrics.

Method	HR			MRR		
	@1	@3	@5	@1	@3	@5
AP-BiLSTM	0.5973	0.9360	0.9688	0.5973	0.7535	0.7613
Ours(g)	0.5978	0.9311	0.9674	0.5978	0.7509	0.7594
Ours(w)	0.5953	0.9292	0.9655	0.5953	0.7491	0.7575
ours	0.7000	1.0000	1.0000	0.7000	0.8433	0.8433

HR@1 and MRR@1: At rank 1, the proposed method (ours) achieves both HR and MRR values of 0.70, significantly outperforming the other methods. This indicates that $70\%$ of the test samples correctly identify the target item at the top of the recommendation list, with an average reciprocal rank of 1.0. These results demonstrate the superior predictive accuracy of the proposed model at the top recommendation position and its effectiveness in identifying the most relevant content for users.

HR@3 and MRR@3: At rank 3, the proposed method achieves an HR of 1.0000 and an MRR of 0.8433, indicating a $100\%$ hit rate within the top three recommendations and a notably higher average reciprocal rank compared to baseline methods. This further confirms the model’s strong recall ability and effective ranking performance within the top three positions.

HR@5 and MRR@5: At rank 5, the proposed method continues to maintain an HR of 1.0000 and an MRR of 0.8433, demonstrating high stability. Although other methods also achieve relatively high HR values at this rank, their MRR scores remain lower than the proposed method. This indicates that our model not only achieves perfect recall within the top five recommendations but also ensures superior ranking quality, reflecting robust and accurate recommendation performance.

Time Efficiency: Regarding time consumption, the proposed method (ours) demonstrates relatively good efficiency, requiring 191 units of time as shown in Table 3, which is slightly lower than the compared methods. This suggests that the model can achieve a relatively faster training or inference speed while maintaining comparable performance, indicating its practical potential.

**Table 3:** Prediction speed and feature importance (FI) scores in process planning.

Method	AP-BiLSTM	Ours(g)	Ours(w)	Ours
Time	198	196	206	191
F1 score	0.5791	0.5768	0.5720	0.5830

F1 Score Performance: In terms of F1 score, the proposed method achieves a respectable value of 0.5830 (see Table 3), marginally higher than the other methods. This result indicates that the model attains a reasonable balance between precision and recall, showing stable predictive capability. Taken together with the time metric, the model demonstrates some improvement in both efficiency and effectiveness, reflecting promising potential for further optimization.

## 5 Discussion

This paper proposes an Assembly Process Reasoning and Decision-making algorithm based on Bidirectional Long Short-Term Memory (BiLSTM) and attention mechanisms (AP-BiLSTM-ATT), aiming to alleviate, to some extent, common challenges in traditional process planning such as difficulties in knowledge reuse, low efficiency, and slow response times. The approach transforms part attributes, geometric features, and historical process plans into structured data representations, and integrates a BiLSTM network with a multi-head attention mechanism to explore the potential relationships between part features and process plans, thereby better capturing contextual dependencies and semantic weight information. During training, the model learns mappings between features and processes from a large-scale historical process dataset, enabling a basic reasoning and recommendation for new part process plans. We analyzed the attention distribution during model prediction and found it effectively focuses on key features consistent with domain knowledge. For instance, the model assigns higher weights to “surface_roughness” when recommending finishing operations, while paying more attention to “material_hardness” for rough machining decisions. Experimental results indicate that, compared to some traditional approaches, the proposed method demonstrates measurable improvements in terms of accuracy, response speed, and generalization ability, suggesting its potential in complex part process planning tasks. However, this study has limitations that warrant attention. The primary constraint lies in the use of a custom-generated dataset, which may affect generalizability to diverse real-world scenarios. Furthermore, practical deployment challenges such as integration with existing PLM/CAM systems and meeting real-time requirements need addressing. Future work will focus on validation with larger industrial datasets and developing prototype systems for practical implementation.

## Data Availability

Publicly available datasets were analyzed in this study. This data can be found at: xt.peng@sut.edu.cn.
